# Predation of amphibians by carabid beetles of the genus *Epomis* found in the central coastal plain of Israel

**DOI:** 10.3897/zookeys.100.1526

**Published:** 2011-05-20

**Authors:** Gil Wizen, Avital Gasith

**Affiliations:** Department of Zoology, Tel-Aviv University, Tel-Aviv 69978, Israel

**Keywords:** *Epomis*, Carabidae, amphibians, predation, feeding behavior, congeneric difference in food habit

## Abstract

The genus *Epomis* is represented in Israel by two species: *Epomis dejeani* and *Epomis circumscriptus*. In the central coastal plain these species are sympatric but do not occur in the same sites. The objective of this study was to record and describe trophic interactions between the adult beetles and amphibian species occurring in the central coastal plain of Israel. Day and night surveys at three sites, as well as controlled laboratory experiments were conducted for studying beetle-amphibian trophic interaction. In the field we recorded three cases of *Epomis dejeani* preying upon amphibian metamorphs and also found that *Epomis* adults share shelters with amphibians. Laboratory experiments supported the observations that both *Epomis* species can prey on amphibians. Predation of the three anuran species (*Bufo viridis*, *Hyla savignyi* and *Rana bedriagae*) and two urodele species (*Triturus vittatus* and *Salamandra salamandra infraimmaculata*) is described. Only *Epomis dejeani* consumed *Triturus vittatus*. Therefore, we conclude that the two species display a partial overlap in food habit.

## Introduction

Invertebrates are known predators of juvenile and adult amphibians. The majority of reports list arachnids (e.g. Formanowicz et al. 1981; McCormick and Polis 1982; Dehling 2007) and aquatic hemipterans (e.g. Hinshaw and Sullivan 1990; Haddad and Bastos 1997; Toledo 2005) as the main arthropod predators. A few studies report predation by ants (Freed and Neitman 1988; Zuffi 2001; Ward-Fear et al. 2009) and by adult beetles (McCormick and Polis 1982; Hinshaw and Sullivan 1990; Jung et al. 2000). The latter involves mostly carabid beetles (Littlejohn and Wainer 1978; Ovaska and Smith 1988; Robertson 1989).

Following Brandmayr et al. (2010) we rank *Epomis* as a separate genus and not as a subgenus of *Chlaenius*. The genus *Epomis* belongs to the Chlaeniini tribe in which about 20 species are known, mainly from tropical Africa and south and south-eastern Asia. Five species are known from the Palaearctic region (Kryzhanovskij 1983).

So far, to the best of our knowledge, predation of an amphibian by an adult *Epomis* beetle was reported in a single note, describing the predation of a juvenile *Rana nigromaculata* by *Epomis* *nigricans* Wiedemann 1821, in Japan (Toshiaki 2006). Recently, predation of juveniles of two amphibian species (*Bufo viridis* and *Hyla savignyi*) by larvae of the carabid beetle *Epomis dejeani* Dejean & Boisduval 1830, was reported (Elron et al. 2007). Until 2007 only *Epomis dejeani* was known from Israel (Elron et al. 2007); however, while conducting this study we discovered an additional species , *Epomis circumscriptus* Duftschmid 1812 (identified by Pietro Brandmayr). In the central coastal plain we found the *Epomis* beetles in clay type and sandy soils around the banks of rain-pools (Elron et al. 2007). Rain-pool habitats are the major breeding sites of amphibians in Israel. Here we report on the food habit and predation behavior of adults of the two *Epomis* species in Israel.

## Methods

### Distribution

During the period of 2007 – 2009 we conducted 103 daytime surveys at 26 sites along the central coastal plain (from south of Tel-Aviv to north of Hadera) in order to examine the presence of *Epomis* species close to freshwater bodies where amphibians are usually present. The specimens observed were identified and recorded. Selected specimens were deposited in the Natural History Collection, Tel-Aviv University.

### Field observations

**Figure 1.**
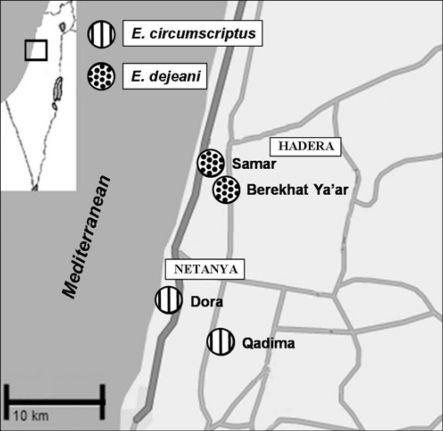
Distribution of *Epomis* species in the study area, central coastal plain, Israel, 2007–2009 (square in left corner shows location of study area).

We conducted daytime and night surveys at three sites in the central coastal plain (Table 1). The location of the study sites is shown in Figure 1. Outside this study, observations on *Epomis circumscriptus* life history dynamics were conducted in two additional sites in the central coastal plain (Qadima and Kfar Netter, Table 1).

**Table 1.** Location and number of daytime and night surveys conducted in the study sites.

*Site name*	*Coordinates*	*daytime surveys*	*night surveys*
Dora	32°17'30"N, 34°50'48"E	27	5
Berekhat Ya’ar	32°24'16"N, 34°54'61"E	37	11
Samar	32°26'23"N, 34°53'01"E	15	11
Qadima	32°27'25"N, 34°89'64"E	-	-
Kfar Netter	32°28'65"N, 34°87'28"E	-	-

During daytime surveys we searched for adult beetles under natural and artificial shelters. The former consisted of any local wooden debris or rocks of various sizes. For artificial shelters we used 40×40 cm cement tiles. At night we used white-light flashlights (Hyundai, Search Finder 1×106 candle power) to locate adult beetles and amphibians and to record their activity outside shelters. Each survey (day or night) lasted for two hours. When predation interaction was encountered, the entire event was recorded.

### Laboratory observations

We supplemented the field observations of predation interactions with controlled experiments in the laboratory, in which we exposed a known species of amphibian to one or other species of *Epomis*. The encounter experiments were conducted in one liter plastic containers (10.5cm high; 14.5cm diameter) with moist peat-moss as substrate in which an individual beetle was reared. A randomly selected metamorph of one out of five amphibian species occurring in the coastal plain was added to the container with the beetle. These metamorphs were measured (snout-vent for anurans; snout-end of tail for urodeles) with a caliper (± 0.05mm) and weighed using an analytical scale (± 0.001g). For each experiment we used a naive amphibian and beetle. Beetles presented with crushed house crickets (*Acheta domestica*) served as a control for feeding interaction. The beetles are used to this food because we routinely feed them with crushed crickets once a week. We fed the amphibian metamorphs daily with live house crickets. Food was not presented to the beetle or the amphibian on the day of the experiment. All observations were made under natural light. We documented the predation encounter using a Canon powershot SX10 video camera. The video recording started 10 seconds before releasing the amphibian into the beetle’s container, and was carried out in 10 minute clips until the interaction ended. In addition, we documented the interaction with still photographs (DSLR, Canon EOS 20D and Canon EOS 50D).Distribution records and observations of predation behavior did not require statistical analysis.

## Results

### Distribution

In 103 surveys conducted in 26 sites in the coastal plain, *Epomis* beetles were recorded in four sites only, all within a radius of 18km (Table 2). The two species were never found in the same site (Fig. 1); *Epomis dejeani* was found in Berekhat Ya’ar and Samar, whereas *Epomis circumscriptus* was found in Dora, Qadima and Kefar Netter (west of Qadima).

**Table 2.** Distances (in km) between the surveyed sites, central coastal plain, Israel.

	*Dora*	*Qadima*	*Berekhat Ya’ar*	*Samar*
Dora	-			
Qadima	5.1	-		
Berekhat Ya’ar	14.4	14.8	-	
Samar	16.3	17.4	2.8	-

### Field observations

We observed three events of adult beetles, *Epomis dejeani* only, preying on *Bufo viridis* metamorphs (two in March, one in July), all during night surveys. On seven out of 79 daytime surveys we recorded adult beetles co-occurring with amphibians (metamorphs, juveniles and an adult) under the same shelters (Table 3; URL: Amphibian - Adult *Epomis* interaction). In all these cases a single adult beetle (male or female) was sharing a shelter with amphibians. Co-occurrence with *Epomis circumscriptus* was recorded in March and April and with *Epomis dejeani* in February, March and May. Although we did not observe predation interaction in the above cases we did find in one case the remains of three devoured metamorphs of *Bufo viridis* (URL: Amphibian - Adult *Epomis* interaction). One of the authors observed similar remains of *Bufo viridis* under a shelter occupied by *Epomis circumscriptus* at another site (Qadima, Fig. 1).

**Table 3.** Developmental stage and number of individuals of amphibians (Adl.= Adult; Juv.= Juvenile; Met.= Metamorph; in parentheses, number of records) recorded co-occurring with adult *Epomis* beetles in the field under the same shelter.

	*Epomis circumscriptus*			*Epomis dejeani*		
Amphibian species	Adl.	Juv.	Met.	Adl.	Juv.	Met.
*Bufo viridis*	0 (45)	1 (2)	30 (1)	0 (72)	0 (72)	0 (72)
*Hyla savignyi*	0 (45)	0 (45)	0 (45)	1 (1)	1 (1)	2 (1)
*Rana bedriagae*	0 (45)	0 (45)	4 (1)	0 (72)	0 (72)	0 (72)

### Laboratory experiments

In the laboratory we found that *Epomis dejeani* preyed on all five amphibian species presented to it in 38 experiments (100% predation occurrence, Table 4). In the case of *Epomis circumscriptus* predation occurred in 78% of 37 experiments. In all the experiments involving *Triturus vittatus* and *Epomis circumscriptus*, predation did not take place (Table 4).

**Table 4.** Comparison of predation of juveniles of five amphibian species by adult beetles of two *Epomis* species. Weights and lengths (anurans – snout-vent; urodeles - snout-end of tail) of the amphibians and shown. n indicates number of experiments.

*Amphibian species*	*Mean weight ±SD (g)*	*Mean length ±SD (mm)*	*Epomis circumscriptus*		*Epomis dejeani*	
			*Predation (%)*	*n*	*Predation (%)*	*n*
*Bufo viridis*	0.38**±**0.11	16.3**±**1.5	100	17	100	18
*Hyla savignyi*	0.24**±**0.03	15.8**±**1.0	100	5	100	5
*Rana bedriagae*	1.24**±**0.32	23.4**±**1.4	100	5	100	5
*Triturus vittatus*	0.21**±**0.03	33.0**±**1.9	0	8	100	8
*Salamandra salamandra infraimmaculata*	1.19**±**0.36	54.7**±**4.1	100	2	100	2

### Predation behavior

On March 26th, 2008 at ca. 10 pm we observed at the Berekhat Ya’ar site, ca. 50m from the pond, an *Epomis dejeani* female biting a *Bufo viridis* metamorph on the lower back area and dragging it for a short distance (ca. 20cm). We then observed the female devouring the metamorph for a period of 27 minutes, starting at the back area, and leaving only the fore and hind limbs. Twenty minutes later, at a distance of ca. 250m from the pond, we observed a different *Epomis dejeani* female feeding on a *Bufo viridis* metamorph in a crevice in the ground. On July 6th, 2008 at 7 pm we observed on the pond bank at the Samar site a male *Epomis dejeani* feeding on a *Bufo viridis* metamorph. The beetle was chewing on the rear legs of the metamorph. Upon our approach it abandoned the site, leaving its prey behind.

**Figure 2.**
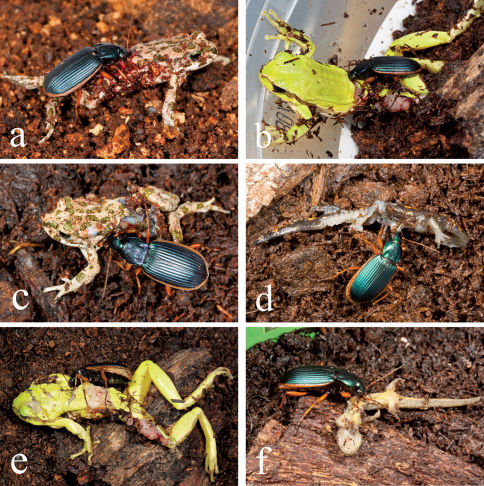
Predation of amphibians by adult *Epomis*: **a** *Bufo viridis* juvenile by *Epomis circumscriptus* **b** *Hyla savignyi* juvenileby *Epomis circumscriptus* **c** *Bufo viridis* juvenile by *Epomis circumscriptus* **d** *Salamandra salamandra infraimmaculata* metamorph by *Epomis dejeani* **e** *Hyla savignyi* juvenile by *Epomis circumscriptus* **f** *Triturus vittatus* metamorph by *Epomis dejeani* (photographs by Gil Wizen).

In all of the laboratory experiments involving *Bufo viridis*, *Hyla savignyi* and *Salamandra salamandra infraimmaculata* metamorphs, adults of both *Epomis* species demonstrated a similar response of immediately jumping on the amphibian’s back, biting at the lower back area (Fig. 2a). This caused the amphibian metamorph to jump, trying unsuccessfully to shake the beetle off. Using its mandibles, the beetles made a horizontal incision in the lower back of the amphibian (Fig. 2b) causing it to cease moving within ca. 1–2 minutes. Subsequently the beetle started chewing on the back and sides of the metamorph (Fig. 2c). Within an hour (*Hyla savignyi* and *Salamandra salamandra infraimmaculata*) to an hour and a half (*Bufo viridis*), only the amphibian’s limbs and head remained (Fig. 2d). In all these cases the beetle’s abdomen swelled noticeably (Fig 2e). In some cases (*Bufo viridis* n=5; *Hyla savignyi* n=4; *Salamandra salamandra infarimmaculata* n=2) the beetle continued feeding, consuming the amphibian’s eyes as well. In all cases (n=5 for *Epomis dejeani*; n=5 for *Epomis circumscriptus*), predation of *Rana bedriagae* metamorphs started with the beetle biting at one of the rear limbs. Despite the vigorous jumping of the *Rana* metamorph the beetle hung on successfully. Within ca. 40 seconds the metamorph ceased to struggle and the beetle changed position to the posterior venter where it initiated chewing. Feeding continued for ca. two hours.

Four out of the five amphibian species were consumed by the two *Epomis* species, whereas *Triturus vittatus* was consumed only by *Epomis dejeani*. In all cases, predation of *Triturus vittatus* started by biting at the central venter (Fig. 2f). Feeding lasted for 27–34 minutes, and when it ended only a few bones remained. In contrast, most *Epomis circumscriptus* (n=5) completely avoided any encounter with *Triturus vittatus*. In two cases of *Epomis circumscriptus* the beetle jumped on the newt but did not initiate biting, and within ca. 10 seconds turned away from the amphibian. It then moved its forelegs and antennae through its mouth parts; this display appeared as cleaning behavior. In one case *Epomis circumscriptus* clasped *Triturus vittatus* by its neck using its mandibles and carried it for a short distance (ca. 10cm). The beetle then dropped the newt on the ground and ceased biting. The beetle was restless, repeatedly moving its forelegs and antennae through its mouth parts as described above.

The amphibian-*Epomis* predation interaction is demonstrated in photos and short videos (URL: Amphibian - Adult *Epomis* interaction).

## Discussion

Two *Epomis* species occur in the central coastal plain of Israel. In the course of this study, they were recorded in four sites only, within a radius of <20 km, but never in the same site. Climate, soil type and vegetation were similar in the four sites in which the beetles occur. In the absence of neither a physical barrier nor an apparent habitat difference the segregation of the species to different sites may be a case of sympatric species that do not occur in the same sites (reviewed in Fitzpatrick et al. 2008). Except for a single observation from 1927 (O. Theodor) where the two species were collected at Hadera (no site information), sympatric distribution with no overlap is supported by all other records of the Natural History Collection, Tel-Aviv University.

Adults of the two *Epomis* species share shelters with amphibians during the day. The encounter between predator and prey is inevitable when the two become active at night. The outcome of this interaction is invariably fatal for the amphibian. Adult Carabidae are phytophagous, zoophagous and mixophagous (Kryzhanovskij 1983). The diet of predacious carabids is diverse, including insects, arachnids, gastropods, isopods and lumbricid worms (Lövei and Sunderland 1996), as well as injured and dead vertebrates (Littlejohn and Wainer 1978). Adult beetles of the Chlaeniini tribe are known to feed on various live and dead invertebrates as well as on carcasses of vertebrates (Kryzhanovskij 1983). The diet of *Epomis* species corresponds to the Chlaeniini food habit, with the addition of live amphibians as an optional food item in their diet. We examined *Epomis* interactions with five out of six amphibian species occurring in Israel. We avoided using the anuran *Pelobates syriacus* which is a rare species in Israel. The beetles’ interaction with this species awaits examination. We describe the predation behavior of the two *Epomis* species based on laboratory observations. The behavior agrees with that described for *Epomis nigricans* in the field (Toshiaki 2006). Nevertheless, further observations in the field are required to support our laboratory observations.

In the field we have evidence for predation of *Bufo viridis* by the two *Epomis* species. In laboratory experiments we found that one of the *Epomis* species preyed upon three anurans and two urodeles while the other species avoided *Triturus vittatus*.

An in-depth investigation of predation of amphibians by *Epomis* species in Israel has revealed that the diet of the two sympatric congeners that do not occur at the same site overlaps only partially. Most reported studies on food habits demonstrate diet partitioning as well as overlap in congeneric sympatric species. These reports include vertebrates such as fish (Targett 1978; Yang and Livingston 1986; Correra et al. 2009), amphibians (Fraser 1976; Dolmen and Koksvik 1983; Griffiths 1986), reptiles (Rose 1976), birds (Schoener 1965; Holmes and Pitelka 1968), and bats (Arlettaz et al. 1997; Lopez and Vaughan 2007). Relatively little is known on food habits of sympatric congeneric insects, such as herbivorous insects (Janz and Nylin 2008), predacious hemipterans (Anderson 1962), herbivorous coleopterans (Futuyma and Mitter 1996), lepidopterans (Chew and Renwick 1995; Menken 1996; Friberg and Wiklund 2008) and hymenopterans (Heatwole and Davis 1965). Most of the reports on insects discuss food overlap (e.g. Futuyma and Mitter 1996; Friberg and Wiklund 2008), and only a few deal with congeneric species with a specialized diet (e.g. Heatwole and Davis 1965; Chew and Renwick 1995; Menken 1996). Among congeneric predacious adult insects that exhibit sympatric distribution but do not occur in the same site, we know of no other example of partial food overlap other than the *Epomis* species we studied. The reason for the partial overlap in the two *Epomis* species is still unknown. A possibility of anti-predator defensive mechanism seems less probable because the known defense responses of amphibian are not species specific (reviewed in Dodd 1976 and Dodd and Brodie 1976). Presently, we examine whether the same difference in food habit found for the adult beetles holds for the larval stages as well.
